# Astrocytic–Neuronal–Astrocytic Pathway Selection for Formation and Degradation of Glutamate/GABA

**DOI:** 10.3389/fendo.2014.00042

**Published:** 2014-04-03

**Authors:** Leif Hertz, Tiago B. Rodrigues

**Affiliations:** ^1^College of Basic Medical Sciences, China Medical University, Shenyang, China; ^2^CRUK Cambridge Institute and Department of Biochemistry, University of Cambridge, Cambridge, UK

**Keywords:** aminoacid transporters, appetite regulation, astrocyte–oligodendrocyte interaction, astrocytic gene expression, brain ammonia, brain aminoacids, brain metabolism, pancreatic islets

Endocrinological research early recognized the importance of intercellular interactions, initially in processes involved in lactation, pubertal maturation, and regulation of the female ovarian cycle and later in appetite regulation. The importance of glutamatergic and GABAergic signaling during all of these events is now realized. Reference (1) describes existing knowledge of the role of amino acid neurotransmitters in the mechanism of neuronal activation during appetite regulation and associated neuronal–astrocytic metabolic coupling mechanisms. Different responses in these mechanisms are apparently originated in different feeding paradigms associated with appetite stimulation (1).

Formation of transmitters glutamate and GABA requires profound interactions between neurons and astrocytes, as does resupply of released transmitters. Both of these amino acid transmitters are formed in brain from glucose in astrocytes (2, 3), but not in neurons, which lack the enzyme pyruvate carboxylase (PC). The most recent progress in measurement of brain glucose transport and metabolism *in vivo* and its importance for understanding of the glial role in glutamatergic and GABAergic neurons are reviewed in Ref. (4), which also thoroughly describes different approaches to establish mathematical models of brain metabolism and apply them to obtain quantitative metabolic rates (4).

**Figure 1:**
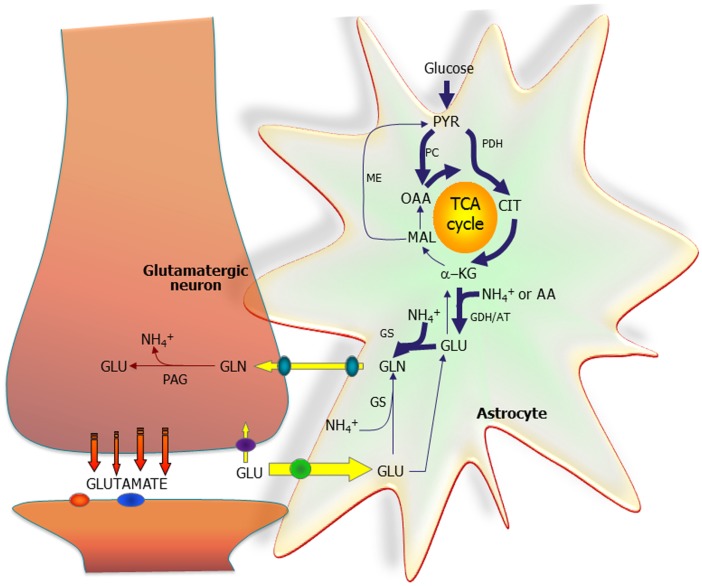
**The astrocytic part of the synapse provides net synthesis of glutamine (GLN), via the concerted action of pyruvate carboxylase (PC) and pyruvate dehydrogenase (PDH), generating oxaloacetate (OAA) and acetyl-CoA, the combination of which leads to synthesis of citrate (CIT)**. This subsequently leads to a net synthesis of α-ketoglutarate (α-KG) allowing synthesis of glutamate (GLU), catalyzed by either glutamate dehydrogenase (GDH) or an amino acid aminotransferase (AA). GLU is used for synthesis of GLN catalyzed by glutamine synthetase (GS). GLN is transferred to the glutamatergic neuron to be used for synthesis of GLU catalyzed by phosphate-activated glutaminase (PAG). Released GLU is taken up into the astrocyte and transformed into GLN completing the GLU–GLN cycle. Alternatively, the GLU taken up may be oxidatively metabolized, which subsequently requires *de novo* synthesis of GLN via the anaplerotic processes indicated in bold arrows. Reproduced from Ref. (2), which together with other contributions discusses metabolic interactions in detail, even in the brain *in vivo*. However, the Figure also shows that NH_4_^+^ is required in astrocytes and released in neurons, and mechanisms transferring NH_4_^+^/NH_3_ between the two cell types are discussed in other articles. So are the transporters releasing glutamine from astrocytes and accumulating it in neurons and the powerful transporters accumulating glutamate in astrocytes, as well as associations between glutamate uptake and metabolism. AT, aminotransferase; MAL, malate; ME, malic enzyme; PYR, pyruvate. Figure from Schousboe et al. (2).

Figure 1 shows that both PC and pyruvate dehydrogenase are needed to form a new molecule of the tricarboxylic acid (TCA) cycle constituent citrate, from which glutamate is generated via α-ketoglutarate. An important, debated question is whether this process is catalyzed by glutamate dehydrogenase (GDH), as generally assumed, or by aspartate aminotransferase (3, 5), suggested by a large stimulation of glutamate/glutamine formation in astrocytes in the presence of aspartate (5). The latter concept is consistent with extremely high cytosolic and mitochondrial aspartate aminotransferase activity, allowing rapid nitrogen exchange between glutamate and aspartate (6).

Glutamate is converted to glutamine by glutamine synthetase (GS) and transferred to neurons. In glutamatergic neurons, glutamine is converted to glutamate within the mitochondrial membrane, enters the mitochondrial matrix, and is returned to the cytoplasm in a process requiring the malate–aspartate cycle operation (2, 3). GABA formation is slightly more complex, since part of the glutamate – from which GABA is formed by decarboxylation – is treated similarly, but another major part is first partly metabolized via the TCA cycle (2). Maximal glutamate synthesis rates in rats/mice are not achieved until postnatal day 30 (3), associated with huge increases in energy demand and production, and probably with functional gains. After neuronal glutamate or GABA release, some transmitter, mainly GABA, is reaccumulated into neurons but most glutamate is returned to astrocytes. Here a part is oxidized, requiring similar *de novo* synthesis to maintain mass balance, and the remainder is returned to neurons for reuse. Both processes are probably identical in brain (2, 3) and in retina (7), where Müller cells are the major glial cells. They express PC (8) and may synthesize glutamate/GABA like brain astrocytes. By removing most extracellular glutamate in the inner retina and contributing to glutamate clearance around photoreceptor terminals, they contribute to shape (and terminate) synaptic activity (7). Reactive Müller cells are neuroprotective, but may also contribute to neuronal degeneration by reversal of glial glutamate transporters. Dysregulation of retinal glutamate homeostasis is important in many retinopathies. A hormonally induced increase in Müller cell GS protects against neuronal injury, whereas GS inhibition increases cell death (7, 9). The possibility that oxidation of astrocytically generated glutamate represents a major part of astrocyte energy metabolism (3) might contribute to this.

Synthesis of pyruvate from glucose involves one oxidative reaction, leading to cytosolic formation of NADH from NAD^+^. For regeneration of NAD^+^, reducing equivalents must be transferred to mitochondria. In brain, this is generally supposed to occur via the malate–aspartate shuttle. Immunohistochemical expression of aralar, an essential constituent of this shuttle, is low in astrocytes (5), but determination of mRNA for its gene in freshly obtained astrocytes and neurons shows equal expression in each cell type (3, 10). A study of the ability of different techniques to demonstrate gene expression in astrocytes showed that a multitude of astrocytic genes, including aralar, seem almost impossible to demonstrate by immunohistochemistry/*in situ* hybridization. Unfortunately, astrocytic gene expression is also occasionally missed by newer microarray studies. Another study evaluated data for GS expression (11), a key enzyme in glutamate/GABA synthesis (Figure 1) first shown immunohistochemically in Ref. (12). Anti-GS was concluded to be the most general astrocytic marker, covering all astrocytic subtypes, and labeling astrocytic cells but no other cell types *in situ*, in culture or in tumors (11). In spite of several reports to the contrary, anti-GS does not label oligodendrocytes, emphasizing the difficulty of evaluation of cellular localization and the importance of cell-specific features for histological verification. Nevertheless, interactions between oligodendrocytes, astrocytes, and neurons are important for many aspects of brain function (13). It is essential to obtain more information about these basic metabolic interactions, which remain under-studied in spite of the importance of white matter disease. Vesicular release of glutamate occurs in white matter, cells of the oligodendrocytic lineage express glutamate receptors, and oligodendrocytic glutamate toxicity is co-implicated in hypoxic–ischemic, inflammatory, and traumatic brain damage (13). Involvement of astrocytes in white matter disease is also shown in tissue from patients having suffered from multiple sclerosis, through the absence of β_1_-adrenergic receptor, and has potentially wide-ranging consequences (14). Moreover, a normal metabolic response to highly elevated K^+^ concentrations is absent in cultured astrocytes from the convulsing Jimpy mice (15).

Glutamine exit from astrocytes and entry into neurons are of equal importance to glutamine synthesis for regulation of *de novo* synthesis of glutamate/GABA and for the return of released transmitter via astrocytes to neurons. The system N transporter SN1 resides on perisynaptic astroglial cell membranes and mediates electroneutral and bidirectional glutamine transport (16). Its activity is regulated at many levels, e.g., by extracellular pH, because protons compete with Na^+^ required for its transport activity. There are consistent observations that SN1 is down-regulated by protein kinase C phosphorylation, probably by internalization (16). Secretion of insulin and glucagon from pancreatic islets resembles other endocrine secretions in their glutamate and GABA dependence, but an even closer resemblance with brain cells is revealed by expression of similar transport processes (17). Islet β- and α-cells contain high levels of glutamate, GABA, and glutamine and their respective vesicular and plasma membrane transporters, which may play important roles in hormone maturation and secretion. Dependent upon secretion needs, glutamine may enter or leave β-cells via SN1 and be taken up by α-cells by SAT2, one of the SAT isoforms that accumulates glutamine in neurons (17).

Since both glutamate and GABA cycles require ammonia fixation in the astrocytic cytosol, and glutamine deamidation to glutamate in neurons, ammonia shortage occurs in the astrocytic, and ammonia excess in the neuronal cytosol (6, 18). This imbalance requires that excess ammonia in neurons either diffuses via the extracellular space to the astrocyte, probably as NH_3_, or that it diffuses into mitochondria, becomes fixed to α-ketoglutarate, and forms glutamate, from which ammonia is returned to astrocytes through the aid of amino acid shuttles. Both Ref. (18), an advanced statistical computational model, and Ref. (6), discussing experimental observations, consider the requirement of this process for neuronal GDH to run in its reductive direction as evidence against its occurrence. This and Ref. (19) contradict a previously suggested major role of branched-chain amino acids or alanine shuttles. However, it is suggested that leucine, which enters the brain from the circulation, might supplement glutamine as an astrocytic–neuronal nitrogen carrier (18).

Glutamatergic and GABAergic activity is terminated by cellular uptake (20). The various transporters have different properties and different regulatory mechanisms, and some also act as ion channels. To understand the physiological roles of the individual transporter subtypes, their anatomical distribution must be known. Quantitative information about the expression is essential since functional capacity is determined by the number of transporter molecules. The most important and most abundant transporters for removal of transmitter glutamate in the brain are EAAT2 (GLT-1) and EAAT1 (GLAST), which both catalyze rapid uptake into astrocytes. GAT1 and GAT3 are the major GABA transporters in the brain, with GAT3 being astrocyte-specific.

Inhibition of GDH-mediated glutamate conversion to α-ketoglutarate with any of three inhibitors (epigallocatechin–monogallate, hexachlorophene, and bithionol) impedes glutamate uptake in the brain through cortical membranes expressing GLT-1 (21). This is consistent with this group’s previous observations of anatomical and physical linkages between astrocytic glutamate transporters and mitochondria. The inhibitors had no effect in cerebellar membranes, where glutamate is accumulated by GLAST, but they did inhibit GABA uptake, suggesting that the GDH plays a role also in GABA metabolism. GABA enters the TCA cycle via succinate and succinic semialdehyde, but glutamate is required if the succinic semialdehyde formation occurs by transamination (3).

The high rate of glutamate uptake (20) together with the close association between glutamate uptake and metabolism (21) suggests that glutamate must be metabolized at high rates in astrocytes. This is convincingly shown in a review (22), pointing to several studies showing that glutamate uptake in astrocytes is more than high enough to meet the demand for its own energy-consuming uptake, and providing an excellent illustration of the metabolic processes in which ATP is generated. They include those involved in complete oxidation of malate via pyruvate recycling and the cytosolic enzyme malic enzyme (ME) (Figure 1). Both Ref. (21) and (22) assume that the initial conversion of glutamate to α-ketoglutarate is mediated by GDH, as always found with isolated cells. However, Balazs found that transaminase-dependent glutamate oxidation accounted for most, but not all, mitochondrial glutamate oxidation (23). Furthermore, in GDH knockout mice most functions remain unchanged (24), except for a reduced glutamate oxidation in cultured, and thus isolated astrocytes. Accordingly, more studies are needed of glutamate/α-ketoglutarate interconversion in intact preparations, a difficult undertaking.
